# Adenosine A_1_ Receptor-Mediated Synaptic Depression in the Developing Hippocampal Area CA2

**DOI:** 10.3389/fnsyn.2020.00021

**Published:** 2020-06-15

**Authors:** Douglas A. Caruana, Serena M. Dudek

**Affiliations:** ^1^School of Life and Health Sciences, Aston University, Birmingham, United Kingdom; ^2^Neurobiology Laboratory, National Institute of Environmental Health Sciences (NIEHS), National Institutes of Health, Research Triangle Park, NC, United States

**Keywords:** adenosine, phosphodiesterase, adenylyl cyclase, synapse, long-term depression (LTD), hippocampus

## Abstract

Immunolabeling for adenosine A_1_ receptors (A_1_Rs) is high in hippocampal area CA2 in adult rats, and the potentiating effects of caffeine or other A_1_R-selective antagonists on synaptic responses are particularly robust at Schaffer collateral synapses in CA2. Interestingly, the pronounced staining for A_1_Rs in CA2 is not apparent until rats are 4 weeks old, suggesting that developmental changes other than receptor distribution underlie the sensitivity of CA2 synapses to A_1_R antagonists in young animals. To evaluate the role of A_1_R-mediated postsynaptic signals at these synapses, we tested whether A_1_R agonists regulate synaptic transmission at Schaffer collateral inputs to CA2 and CA1. We found that the selective A_1_R agonist CCPA caused a lasting depression of synaptic responses in both CA2 and CA1 neurons in slices obtained from juvenile rats (P14), but that the effect was observed only in CA2 in slices prepared from adult animals (~P70). Interestingly, blocking phosphodiesterase activity with rolipram inhibited the CCPA-induced depression in CA1, but not in CA2, indicative of robust phosphodiesterase activity in CA1 neurons. Likewise, synaptic responses in CA2 and CA1 differed in their sensitivity to the adenylyl cyclase activator, forskolin, in that it increased synaptic transmission in CA2, but had little effect in CA1. These findings suggest that the A_1_R-mediated synaptic depression tracks the postnatal development of immunolabeling for A_1_Rs and that the enhanced sensitivity to antagonists in CA2 at young ages is likely due to robust adenylyl cyclase activity and weak phosphodiesterase activity rather than to enrichment of A_1_Rs.

## Introduction

Caffeine acts as a stimulant when consumed by humans, and in some individuals, it may trigger or even exacerbate symptoms of anxiety or psychosis (Lucas et al., 1990; Broderick and Benjamin, 2004). Although the precise cellular mechanisms underlying both the beneficial and detrimental effects of caffeine on cognition remain largely unknown, studies in rodents provide powerful insight into caffeine’s mode of action. Strong evidence links adenosine receptors and in particular the A_1_ receptor (A_1_R) subtype, as major targets of caffeine (Nehlig et al., 1992; Jacobson et al., 1996). Interestingly, mice lacking A_1_Rs show increased aggression and anxiety-like behaviors (Giménez-Llort et al., 2002). Indeed, one brain area that has been associated with social aggression and social recognition memory in hippocampal area CA2 (Hitti and Siegelbaum, 2014; Stevenson and Caldwell, 2014; Pagani et al., 2014). This is notable because pyramidal neurons in CA2 show the highest labeling for A_1_Rs in the entire hippocampus (Ochiishi et al., 1999). Consistent with the high expression of A_1_Rs in CA2 is the observation that caffeine and other A_1_R antagonists preferentially enhance excitatory synaptic transmission in area CA2 at concentrations that have little effect on responses in CA1 and CA3 (Simons et al., 2012). This potentiation differs from typical activity-dependent forms of long-term potentiation (LTP) in that it does not require activation of N-methyl-D-aspartate (NMDA) receptors, a rise in postsynaptic calcium, or activity of Ca^2+^/calmodulin-dependent protein kinase II. It is however, blocked by inhibitors of adenylyl cyclase and postsynaptic protein kinase A (PKA; Simons et al., 2012). Although presynaptic A_1_Rs are known to regulate glutamate exocytosis (Dunwiddie and Hoffer, 1980; Prince and Stevens, 1992; Dunwiddie and Masino, 2001), the effects of caffeine on the release probability of neurotransmitter did not differ between areas CA2 and CA1, suggesting that the CA2-specific potentiating effects of A_1_R antagonists are due to actions mediated mainly by postsynaptic A_1_Rs in CA2 pyramidal neurons (Ochiishi et al., 1999; Simons et al., 2012; but see Muñoz and Solís, 2019).

A difficult finding to reconcile is the observation by Ochiishi et al. (1999) that immunolabeling for the A_1_R is uniform in the pyramidal cell layer across the CA fields in young rats, with virtually no difference in labeling between areas CA2 and CA1 at postnatal day 14, the approximate age of animals used previously by Simons et al. (2012). It is only at older ages—P28 and above—that staining for the A_1_R is most pronounced in CA2 neurons. Curiously, the robust differences between CA2 and CA1 in their ability to support A_1_R-mediated synaptic potentiation do not mirror the developmental expression pattern of the A_1_R observed in young rats. Possible explanations to account for this discrepancy may involve key downstream signaling molecules that regulate either the production (*via* adenylyl cyclases) or degradation (*via* phosphodiesterases) of cyclic adenosine monophosphate (cAMP). Because A_1_Rs couple to G_i/o_ type G proteins to decrease the activity of adenylyl cyclase and constrain the production of cAMP (Fredholm et al., 2011), we assessed whether activation of A_1_Rs would induce synaptic depression in Schaffer collateral inputs to CA2 and CA1. Importantly, we tested whether transmission at these synapses differed in their responses to A_1_R agonists in an age-dependent manner. Finally, we tested whether pharmacological manipulation of the postsynaptic signals recruited by activation of A_1_Rs would unmask differences in synaptic responses evoked in areas CA1 and CA2 in brain slices prepared from juvenile rats, possibly explaining the differences observed between the two subfields in response to an array of A_1_R-selective antagonists, including caffeine.

## Materials and Methods

### Tissue Slices

Methods for obtaining whole-cell voltage-clamp and current-clamp recordings were similar to those described previously (Caruana et al., 2011; Simons et al., 2012; Pagani et al., 2014). Briefly, brain slices were prepared from juvenile (P14–18) male or female Sprague–Dawley rats, as well as from adult males (P60–70; Charles River Laboratories). Animals were anesthetized with sodium pentobarbital (65 mg/kg, i.p.), decapitated and the brains were rapidly removed and transferred into the ice-cold sucrose-substituted artificial cerebrospinal fluid (ACSF) containing (in mM): 240 sucrose, 2.0 KCl, 1 MgCl_2_, 2 MgSO_4_, 1 CaCl_2_, 1.25 NaH_2_PO_4_, 26 NaHCO_3,_ and 10 D-glucose, and saturated with 95% O_2_ and 5% CO_2_. Coronal brain slices (340 μm thick) containing the dorsal hippocampus were taken from sections located within −2.30 and −4.30 mm posterior to Bregma (Paxinos and Watson, 1998). Slices were cut using a vibratome (VT1200S, Leica Biosystems) and then placed in a holding chamber containing normal ACSF (warmed to 32°C), and slices recovered for at least 1 h before experimental recordings. Standard ACSF consisted of the following (in mM): 124 NaCl, 2.5 KCl, 2 MgCl_2_, 2 CaCl_2_, 1.25 NaH_2_PO_4_, 26 NaHCO_3_ and 17 D-glucose. After the recovery period, slices were transferred individually to a recording chamber and visualized using an upright microscope (BX51WI, Olympus Corp.) equipped with differential interference contrast optics, a 40× water-immersion objective and a near-infrared camera (RC300, Dage-MTI). Submerged slices were superfused with oxygenated ACSF at a rate of 2.0 ml/min at room temperature (~25°C).

### Stimulation and Recording

The whole-cell voltage or current-clamp recordings from hippocampal pyramidal neurons were made using patch pipettes filled with a solution containing the following (in mM): 120 K-gluconate, 10 KCl, 3 MgCl_2_, 0.5 EGTA, 40 HEPES, 2 Na_2_-ATP and 0.3 Na-GTP, with pH adjusted to 7.2 by KOH. Electrodes were prepared from borosilicate glass (filamented, 1.5 mm OD; 2.5–3.5 MΩ; King Precision Glass) using a horizontal puller (P-97, Sutter Instrument Co.), and experiments on CA2 neurons were performed only when area CA2 could be distinguished visually from area CA1. Pipettes were placed in contact with somata of visually-identified pyramidal neurons in CA2 and CA1 and gentle suction was applied under voltage clamp to form a tight seal (1–3 GΩ). Whole-cell configuration was achieved by increased suction, and experiments began after cells stabilized (typically within 10–15 min following break-in). Electrophysiological properties of CA2 neurons such as the amplitude of the sag in response to hyperpolarizing currents, capacitance, or resting membrane potentials, while significantly different from neurons in CA1 or CA3 as a group (Zhao et al., 2007 for the rat; San Antonio et al., 2013; Sun et al., 2017 for mice), were varied enough to make them unreliable indicators of CA2 neuron identity due to the large overlap in values. Therefore, the approximate position of CA2 was estimated based on the appearance of the cells (vs. generally unhealthy- appearing CA3 neurons) and position relative to the upper blade of the dentate gyrus. Differences in pharmacology between presumed CA2 neurons and CA1 neurons provided further evidence that we were recording from different populations of neurons.

**Figure 1:**
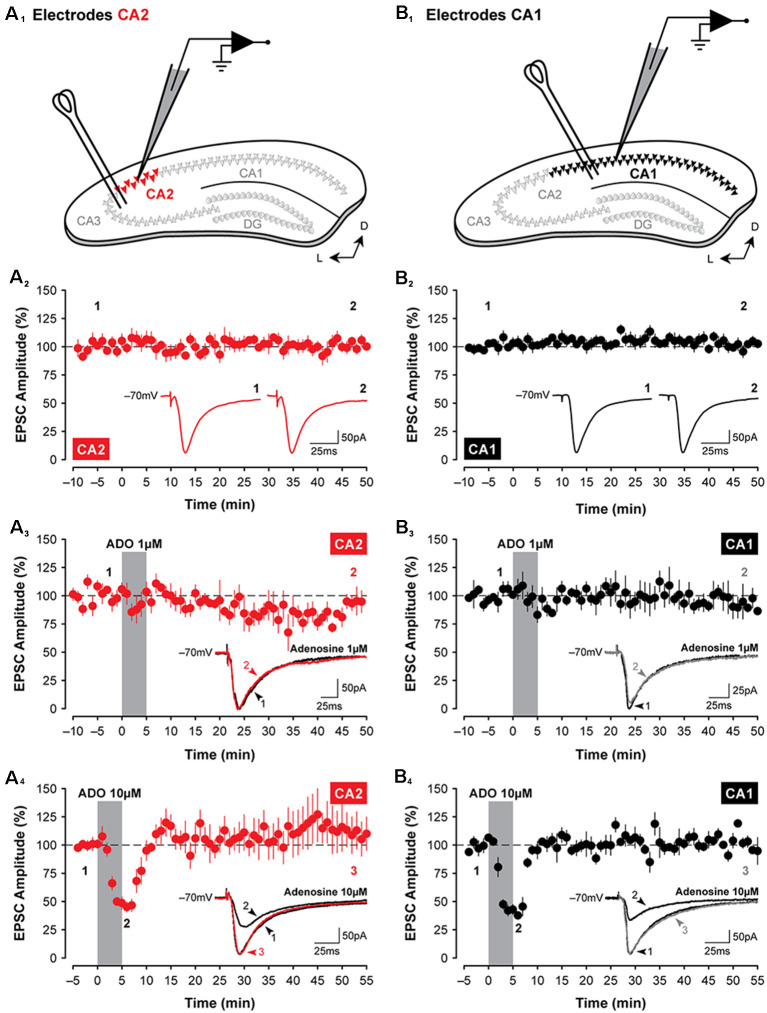
Adenosine has transient suppressive effects on synaptic transmission in hippocampal areas CA2 and CA1. Illustrated are the approximate locations of stimulating electrodes relative to recording electrodes in slices of the dorsal hippocampus (**A_1_**, CA2, in red; **B_1_**, CA1, in black; colors are consistent for each figure). Stimulating electrodes were positioned in the stratum radiatum to activate Shaffer Collateral axons. **(A_2_,B_2_)** Mean (±SEM) excitatory postsynaptic current (EPSC) amplitudes recorded from CA2 and CA1, respectively, for 60 min without treatment. Data from these graphs are plotted in subsequent figures for statistical comparison. The amplitude of EPSCs has been normalized to the pre-treatment baseline for plotting in this and subsequent figures. Inset traces in **(A_2–4_,B_2–4_)** are averaged EPSCs (average of three consecutive sweeps over a 1-min period in this and subsequent figures) from representative experiments at the times indicated by the numbers. **(A_3,4_,B_3,4_)** Bath-application of adenosine (ADO; obtained from Sigma) for 5 min, indicated by the shaded region, induces only a transient suppression of EPSCs in the hippocampus and only at a concentration of 10 μM **(A_4_,B_4_)**. This effect was highly variable and depended on the commercial source of the adenosine (data not shown). Note: recordings were made from slices prepared from juvenile rats (P14 to P18) in this and subsequent figures unless specified otherwise.

Voltage clamp recordings (V_h_ = −70 mV) were obtained using an Axopatch 200B amplifier (Molecular Devices Inc., San Jose, CA, USA) and displayed on a computer monitor using the software package WinLTP (WinLTP Limited). Recordings were filtered at 5 kHz and digitized at 20 kHz (Digidata 1322A, Molecular Devices Inc., San Jose, CA, USA). No correction was applied to compensate for liquid junction potentials. Only cells with a series *resistance* (R_s_) < 25 MΩ and a <20% change in R_s_ from baseline during an experiment were included for analysis. Synaptic responses in CA2 and CA1 pyramidal neurons were evoked using cluster-style electrodes (CE2C75, FHC Inc.) placed ~150 μm from recorded neurons in the stratum radiatum at a location intended to stimulate the Schaffer collaterals (see Figures 1A_1_,B_1_). Synaptic responses were evoked with 0.1 ms constant current pulses delivered using a stimulus isolation unit (BSI-2A, BAK Electronics) and stimulation intensity was adjusted to evoke synaptic currents approximately 75% of maximal amplitude (range; 75–200 μA). Single test pulses or pairs of stimulation pulses (with a 50 ms interpulse interval) were delivered to the Schaffer collaterals every 20 s to evoke excitatory postsynaptic currents (EPSCs). In some experiments, EPSCs were evoked by stimulation intensities ranging from 10 to 200 μA delivered in ascending steps (input-output tests). Protocols for all synaptic recording experiments were configured and controlled using WinLTP. The intrinsic excitability of pyramidal neurons in CA2 and CA1 was determined by measuring changes in membrane potential following the injection of hyperpolarizing and depolarizing current steps in current-clamp mode using the software package pClamp (Molecular Devices Inc., San Jose, CA, USA).

### Data Analysis

Evoked synaptic currents were analyzed using the software applications WinLTP and AxoGraph X (AxoGraph Scientific). Peak amplitudes of EPSCs were measured relative to the prestimulus baseline (8–2 ms period before stimulation pulse), and paired-pulse facilitation (PPF) was determined by expressing the amplitude of the second response as a proportion of the amplitude of the first response. During synaptic recordings, input resistance (R_in_) was calculated by measuring the amplitude of the steady-state current evoked during a −5 mV voltage step (50 ms duration) delivered 100 ms before test stimulation, and R_s_ was calculated by measuring the peak amplitude of the fast capacitive transient observed at the onset of the voltage step. The coefficient of variation (1/CV^2^; where C = mean and V = standard deviation) was calculated on the mean amplitude of responses obtained during 10 min epochs of stable recordings of EPSCs using Excel (Microsoft Corporation). The stability of responses was first confirmed by testing the slope of a regression line fit through data points included for 1/CV^2^ analyses, and results for 1/CV^2^ analyses were normalized to the baseline for plotting. The effects of bath- or intracellularly-applied drugs during pharmacological experiments were assessed on the amplitude of averaged EPSCs obtained during 5-min-long epochs recorded at different times during an experiment (latencies specified below). Also, experiments were interleaved so that no single experiment was performed twice in slices prepared from the same animal. All data were expressed as the mean ± SEM and were normalized to baseline recordings for plotting. Changes in response properties were assessed with Prism (GraphPad Software Inc.) using (where appropriate) paired or unpaired samples *t*-tests, one-way ANOVAs, or repeated-measures ANOVAs. *Post hoc* comparisons were made using the Bonferroni or Tukey methods with the alpha level set to *P* < 0.05.

The intrinsic excitability of pyramidal neurons was assessed by counting the number of spikes evoked in response to 500 ms-duration suprathreshold depolarizing current steps (0–100 pA in ascending 20 pA increments) from a constant holding potential (typically rest). Input resistance was calculated by measuring both the peak and the steady-state voltage responses to −100 pA current steps (500 ms in duration), and inward rectification was quantified by expressing the peak input resistance as a proportion of the steady-state resistance (rectification ratio). All data were expressed as the mean ± SEM for plotting, and changes in response properties were assessed using paired samples *t*-tests.

### Pharmacology

Unless stated, all compounds used for pharmacological experiments were obtained from Sigma and prepared as concentrated stock solutions (typically 10–50 mM) by dilution in (where appropriate) either distilled water or dimethyl sulfoxide and stored at −20°C until required. Compounds were either bath-applied or loaded intracellularly *via* the patch recording pipette.

## Results

### Effects of Adenosine and an A_1_R Agonist on Synaptic Transmission in CA2 and CA1

EPSCs were evoked in CA2 or CA1 by stimulating the Schaffer collaterals. The schematic diagrams are shown in Figure 1 highlight the placement of stimulating and recording electrodes in coronal slices of the dorsal hippocampus for experiments assessing evoked synaptic responses in area CA2 (Figure 1A_1_) or in area CA1 (Figure 1B_1_). Synaptic responses were stable for 60 min (CA2, Figure 1A_2_; CA1, Figure 1B_2_), and the amplitude of EPSCs did not change significantly during the course of interleaved, age-matched and untreated control experiments (CA2, baseline vs. last 5-min, *t*_(5)_ = 0.11, *P* = 0.92, *n* = 6; CA1, baseline vs. last 5-min, *t*_(6)_ = 0.37, *P* = 0.72, *n* = 7). To determine whether activation of adenosine receptors induces a lasting depression of synaptic responses in CA2 neurons, EPSCs were monitored before, during, and after a brief 5-min-long bath-application of adenosine. We found that although 10 μM adenosine significantly suppressed synaptic transmission during the first 10 min following the onset of drug application (to 51.8 ± 4.8% of baseline, *F*_(1,9)_ = 10.62, *P* < 0.01, Bonferroni *P* < 0.001, *n* = 5; Figure 1A_4_), responses returned to baseline levels quickly during washout and remained stable for the remainder of the experiment (at 110.21 ± 15.31% of baseline during the last 5-min, Bonferroni *P* = 0.88). Unlike the potentiating effects of A_1_R antagonists, which were more pronounced in CA2 (Simons et al., 2012), the effects of adenosine in CA2 did not differ significantly from those in CA1 (compare data shown in Figures 1A_3,4_,B_3,4_). Synaptic responses in Schaffer collateral inputs to CA1 were depressed significantly to 43.19 ± 4.71% of baseline (*F*_(1,9)_ = 89.33, *P* < 0.0001, Bonferroni *P* < 0.0001, *n* = 4, Figure 1B_4_) by 10 μM adenosine and returned quickly to baseline levels during washout (to 102.96 ± 3.01% of baseline, Bonferroni *P* > 0.999). We note, however, that the adenosine-mediated suppression of EPSCs in both CA2 and CA1 was highly variable and depended on the commercial source of the adenosine, with effects ranging from no suppression of EPSCs (using adenosine obtained from Tocris Bioscience; similar to the non-significant results observed using 1 μM adenosine shown for CA2 in Figure 1A_3_, *n* = 4; and for CA1 in Figure 1B_3_, *n* = 4) to brief potentiation (Garaschuk et al., 1992; adenosine from Ascent Scientific; data not shown).

**Figure 2:**
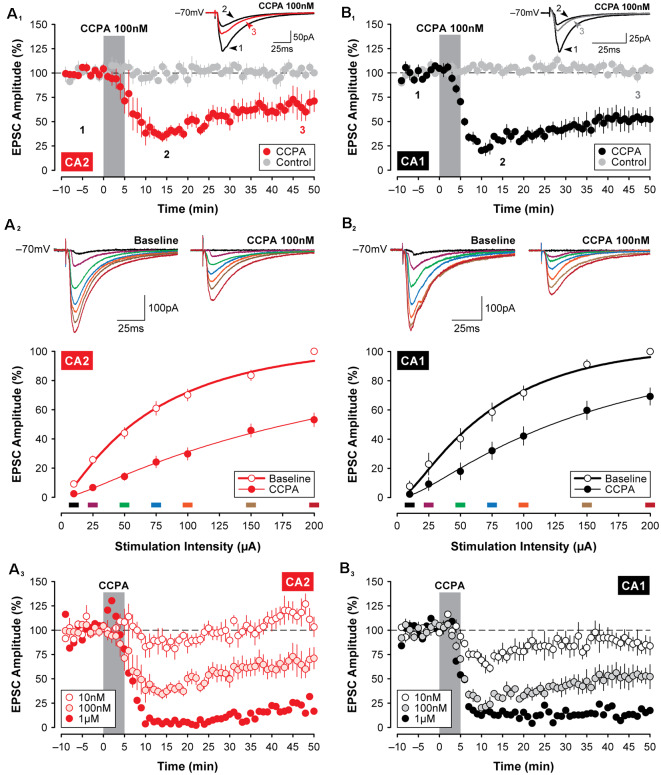
Selective activation of A_1_ receptors (A_1_Rs) with CCPA induces synaptic depression in both CA2 and CA1. Bath application of the A_1_R-selective agonist CCPA (100 nM; gray bar) induced a significant depression in the amplitude of EPSCs in both CA2 **(A_1_)** and CA1 **(B_1_)**. Inset and averaged traces are from a representative experiment at times indicated by the numbers. Recordings from age-matched and untreated control slices are shown in gray for comparison. **(A_2_,B_2_)** The depression of EPSCs induced by 100 nM CCPA is evident across a wide range of stimulation intensities when assessed 15-min into the washout period for area CA2 and CA1, respectively. Inset and colored traces (above) are from representative experiments at the stimulation intensities indicated by the corresponding colored bars (below). **(A_3_,B_3_)** Effects of three doses of CCPA (10 nM, 100 nM, and 1 μM; gray bar) on EPSC amplitudes recorded in CA2 and CA1, respectively. Overall, the effects of CCPA on synaptic function did not differ significantly between areas CA2 and CA1.

Given the variability of the adenosine-mediated effects described above, we reasoned that the stability of adenosine may be problematic and that use of an A_1_R agonist may produce more stable and consistent results. Additionally, an agonist would have the advantage of selectivity for the A_1_R over other adenosine receptor subtypes that are known to be expressed in the hippocampus and would not be subject to degradation by endogenous enzymes. Indeed, a 5-min application of the selective A_1_R agonist 2-chloro-N6-cyclopentyladenosine (CCPA; Lohse et al., 1988; 100 nM; Figure 2A_1_) induced a robust decrease in the amplitude of synaptic responses recorded in CA2 pyramidal neurons, lasting for at least 50 min. EPSCs were depressed significantly to 32.44 ± 4.27% of baseline within the first 15-min of washout relative to age-matched and untreated control responses (*F*_(1,11)_ = 7.84, *P* < 0.05, Bonferroni *P* < 0.0001, *n* = 7, Figure 2B_1_), and EPSCs remained significantly depressed at 61.81 ± 9.38% of baseline by the end of the experiment (Bonferroni *P* < 0.001). The suppression of synaptic responses in CA2 was apparent across a range of stimulation intensities and concentrations of CCPA. The amplitude of synaptic responses in area CA2 increased in proportion to the intensity of electrical stimulation delivered to the Schaffer collaterals, and this was significantly reduced by CCPA when assessed 15 min into the washout period for all but the lowest stimulation intensity tested (*F*_(6,54)_ = 27.72, *P* < 0.001, Bonferroni *P* < 0.001 for 25–200 μA, *n* = 10, Figure 2A_2_). The effect of CCPA on synaptic responses in CA2 was also tested following bath-application of three different concentrations of CCPA (10 nM, *n* = 5; 100 nM, *n* = 7; and 1 μM, *n* = 1), but a depression of EPSCs lasting longer than 15 min was observed only with the two highest concentrations (see Figure 2A_3_).

**Figure 3:**
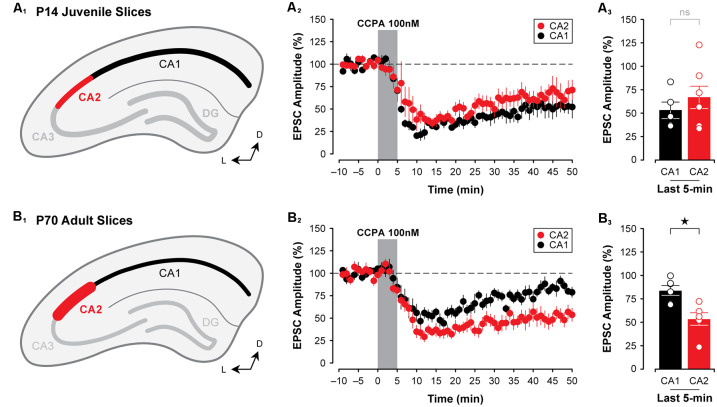
The A_1_R-mediated depression of EPSPs closely mirrors the developmental expression pattern of A_1_Rs in CA2 and CA1. Immunolabeling for A_1_Rs, as reported by Ochiishi et al. (1999); is homogeneous across Ammon’s horn in early postnatal development (depicted in **A_1_**), and A_1_R expression becomes markedly more pronounced in CA2 and weaker in CA1 during adulthood (depicted in **B_1_**). Mirroring this is the observation that the CCPA-mediated depression of EPSCs (100 nM) is similar in both CA1 and CA2 in slices obtained from P14 rats **(A_2,3_)**. Further, the A_1_R-mediated depression induced by CCPA increases with age in CA2, yet decreases with age in CA1 (~P70; **B_2,3_**). *p < 0.05, ns, not significant.

Similar to the short-lasting effects of adenosine shown in Figure 1, the CCPA-mediated depression of EPSCs observed in CA2 did not differ significantly from the depression induced in area CA1 following the same experimental protocols. Bath-application of 100 nM CCPA significantly depressed the amplitude of EPSCs in CA1 to 24.82 ± 3.12% of baseline within the first 15-min of washout relative to age-matched and untreated control responses (*F*_(1,10)_ = 22.32, *P* < 0.001, Bonferroni *P* < 0.0001, *n* = 5, Figure 2B_4_), and EPSCs remained significantly depressed at 52.55 ± 8.59% of baseline by the end of the experiment (Bonferroni *P* < 0.001). And as noted above, the magnitude of the suppression of synaptic responses induced by 100 nM CCPA did not differ between area CA2 and CA1 at the time points assessed (*F*_(1,10)_ = 0.02, *P* = 0.893; peak, Bonferroni *P* = 0.939; last 5-min, Bonferroni *P* = 0.762; data not shown; see also Figures 3A_2,3_). Bath-application of 100 nM CCPA also induced a significant suppression of synaptic responses across a range of stimulation intensities in area CA1 (*F*_(6,48)_ = 5.26, *P* < 0.001, Bonferroni *P* < 0.01 for 25 μA and *P* < 0.0001 for 50–200 μA, *n* = 9, Figure 2B_2_), but interestingly, the magnitude of the suppression was greater in area CA2 than in CA1 at the two highest stimulation intensities tested (*F*_(6,102)_ = 2.51, *P* < 0.05, Bonferroni *P* < 0.05 at 150 and 200 μA, respectively; compare Figures 2A_2_,B_2_). The effect of A_1_R activation on EPSCs in CA1 was also tested following bath-application of multiple concentrations of CCPA (10 nM, *n* = 5; 100 nM, *n* = 5; and 1 μM, *n* = 1), but similar to area CA2, a depression of synaptic responses lasting longer than 15 min was observed only with the two highest concentrations of CCPA (see Figure 2B_3_). Note that we cannot distinguish between the long-lasting biological effects of A_1_R activation and incomplete wash-out of the drug with continuing activation of the receptors. These findings suggest that the effects of selective A_1_R activation, unlike A_1_R blockade (Simons et al., 2012), are similar in CA1 and CA2 neurons at young postnatal ages (P14 to P18; see Figures 3A_2,3_) and closely reflect the developmental expression pattern of A_1_Rs in the hippocampus (see schematic summary in Figure 3A_1_; Ochiishi et al., 1999).

### Developmental Differences in A_1_R-Mediated Synaptic Depression in the Hippocampus

To determine whether the A_1_R-mediated depression of EPSCs differs in CA2 and CA1 at an age when A_1_R expression is known to be highest in area CA2 (Ochiishi et al., 1999; Figure 3B_1_), we tested the effects of 100 nM CCPA on evoked synaptic responses in CA2 and CA1 neurons in slices prepared from adult rats. We found that in slices from adult animals (~P70), CCPA induced a significantly greater depression of synaptic responses in CA2 than in CA1 (*F*_(1,20)_ = 6.37, *P* < 0.05, Figures 3B_2,3_). EPSCs were depressed to 53.57 ± 6.66% of baseline levels in area CA2 (*n* = 6) when assessed at the end of the experiment, whilst responses in CA1 returned to baseline levels (to 83.94 ± 5.12%, *n* = 6; Bonferroni *P* < 0.05, Figures 3B_2,3_). These data suggest that the A_1_R-mediated suppression of EPSCs induced by CCPA closely follows the pattern of immunolabeling for A_1_Rs at both early and late postnatal ages in rats.

**Figure 4:**
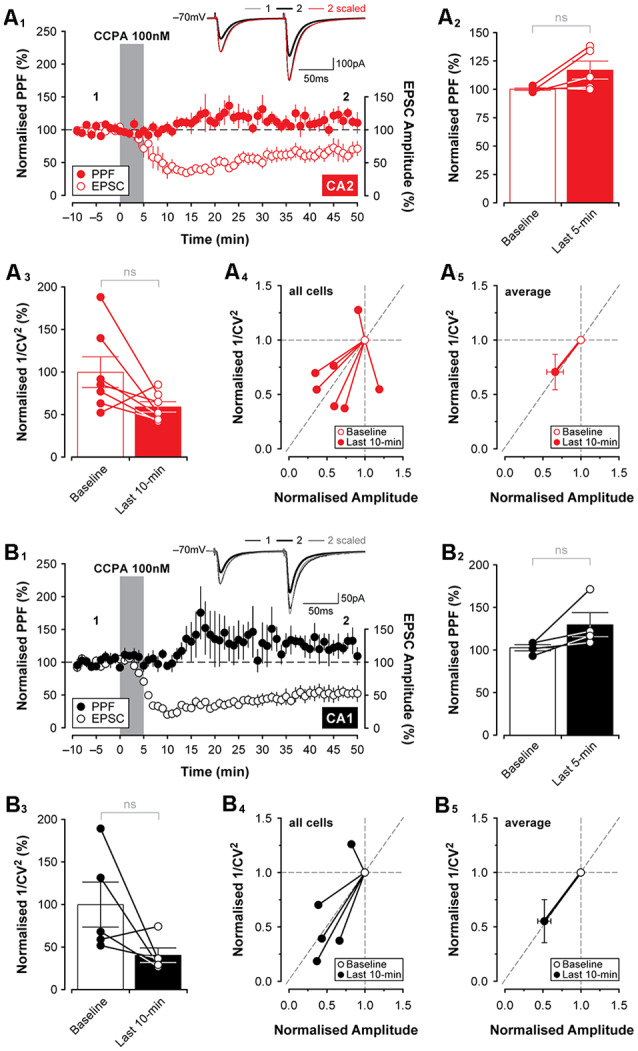
Changes in presynaptic function induced by CCPA do not differ between CA2 and CA1. Paired-pulse facilitation (PPF; 50 ms interpulse interval) did not change significantly following 5-min bath-application of CCPA in area CA2 **(A_1,2_)** or area CA1 **(B_1,2_)**. The CCPA-mediated depression of EPSCs (open circles) is shown together with PPF (filled circles) for illustration purposes in **(A_1_,B_1_**). Inset traces in **(A_1_,B_1_)** are from representative experiments at the times indicated by the numbers. Note that although CCPA reduced the amplitude of synaptic responses, PPF was unchanged when the responses were scaled to the baseline (inset; scaled) in both CA2 and CA1. **(A_3–5_,B_3–5_)** The coefficient of variation (1/CV^2^) was computed for 10-min epochs of stable EPSCs recorded during the baseline period and at the end of the experiment. Bath-application of CCPA increased response-to-response variability calculated during the last 10-min of recording as indicated by a slight reduction in 1/CV^2^ for CA2 **(A_3_)** and CA1 **(B_3_)**, but the reduction was not significantly different from the baseline at either recording site. Normalized 1/CV^2^ for the last 10-min of the experiment is expressed as a ratio of the baseline and plotted against the normalized amplitude of EPSCs for each experiment (CA2, all cells, **A_4_**; CA1, all cells, **B_4_**) and averaged data (CA2, average, **A_5_**; CA1, average, **B_5_**). Results are shown in **(A_5_,B_5_)** indicate that although there is a significant reduction in the amplitude of EPSCs induced by CCPA in CA2 and CA1, respectively, there is no significant change in the variability of evoked responses to suggest whether CCPA was acting on A_1_Rs located pre- or postsynaptically. ns, not significant.

Adenosine, acting on A_1_Rs, has been shown previously to reduce glutamate release (Dunwiddie and Hoffer, 1980; Prince and Stevens, 1992). As such, we assessed whether CCPA was acting presynaptically to induce the depression of excitatory synaptic responses observed in both CA2 and CA1 in slices obtained from young animals. One assessment of presynaptic function that is useful in determining whether changes in synaptic transmission can be attributed to changes in the release probability of glutamate is the test of (PPF; Creager et al., 1980; Zucker, 1989; Zucker and Regehr, 2002). Adenosine has been reported to increase PPF, reflecting a decrease in glutamate release from presynaptic terminals (Harris and Cotman, 1985; Dumas and Foster, 1998; Manita et al., 2007). We found that concurrent with the depression in synaptic responses induced by 100 nM CCPA, PPF (using a 50 ms interpulse interval) increased in some cases. The effect was inconsistent across slices and did not reach statistical significance in either CA2 (increase to 117.0 ± 8.01% of baseline, *t*_(4)_ = 2.14, *P* = 0.099, *n* = 5, Figures 4A_1,2_) or CA1 (increase to 130.1 ± 14.6% of baseline, *t*_(3)_ = 2.13, *P* = 0.122, *n* = 4, Figures 4B_1,2_). An additional measure of presynaptic function that may reflect changes induced by the application of CCPA is the coefficient of variation (1/CV^2^; Caruana et al., 2011; Pagani et al., 2014). Here, 1/CV^2^ was computed for 10-min epochs of stable EPSCs recorded during the baseline period and at the very end of the experiment. Response-to-response variability was increased by CCPA as indicated by a reduction in 1/CV^2^ in both CA2 (to 59.17 ± 6.14% of baseline, *n* = 7, Figure 4A_3_) and CA1 (to 40.48 ± 8.65% of baseline, *n* = 5, Figure 4B_3_), and this suggests that CCPA may have been acting on presynaptic A_1_Rs to suppress glutamate release and regulate synaptic function. However, the reduction in 1/CV^2^ observed at the end of the experiment did not differ significantly from baseline in either CA2 (*t*_(6)_ = 2.23, *P* = 0.066) or CA1 (*t*_(4)_ = 1.95, *P* = 0.123). Data for 1/CV^2^ analyses obtained during the last 10-min of the experiment were also expressed as a ratio of the baseline and plotted against the CCPA-mediated change in the amplitude of EPSCs for each individual experiment (CA2, Figure 4A_4_, all cells; CA1, Figure 4B_4_ all cells), as well as for the pooled data set (CA2, Figure 4A_5_, average; CA1, Figure 4B_5_ average). Results shown in Figures 4A_5_,B_5_ indicate that although we observed a significant reduction in the amplitude of EPSCs induced by CCPA in CA2 and CA1, respectively, we found no significant change in the variability of EPSCs to suggest whether CCPA is acting pre- or postsynaptically. Although these data do not rule out the possibility that the suppression of EPSCs in both CA2 and CA1 resulted, in part, from A_1_R-mediated changes in presynaptic glutamate release, they do argue against a robust involvement of several of the known mechanisms, including regulation of presynaptic calcium, which would have been reflected in the magnitude of PPF observed.

**Figure 5:**
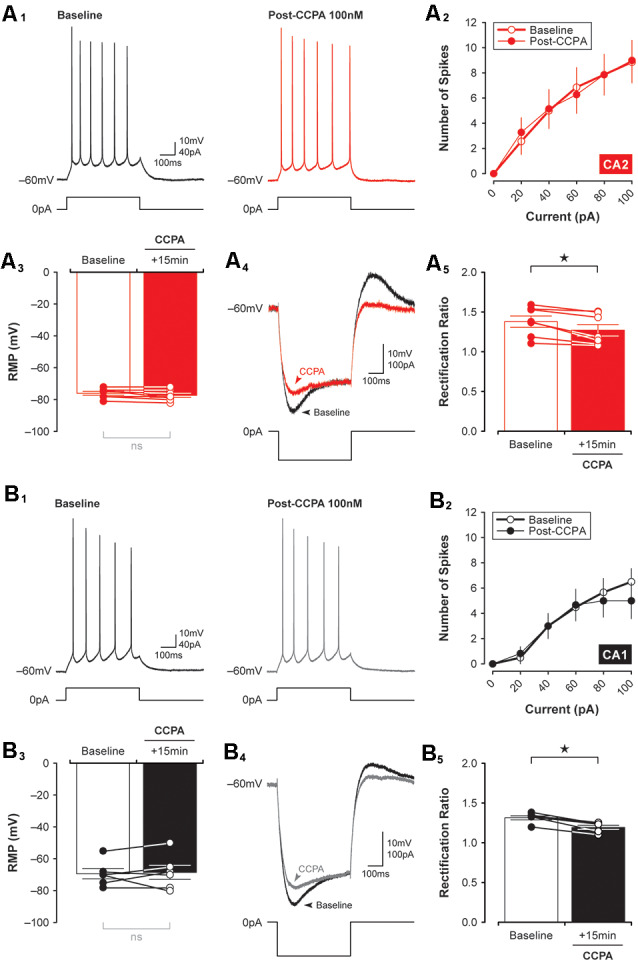
CCPA-mediated changes in inward rectification are similar in both CA2 and CA1. The number of spikes elicited in response to depolarizing current steps was the same in CA2 and CA1 before and 15 min after a brief 5-min-long application of 100 nM CCPA [**A_1_,B_1_**, representative spikes triggered by a +40 pA current step; **(A_2_,B_2_)**, input-output data for spikes initiated by suprathreshold injection of multiple current steps up to 100 pA]. There was also no significant change in the resting membrane potential (RMP) of neurons recorded in CA2 **(A_3_)** and CA1 **(B_3_)** before and after CCPA. Inward rectification was quantified by expressing the peak input resistance as a proportion of the steady-state input resistance (rectification ratio). Rectification ratios decreased significantly following CCPA treatment in both CA2 **(A_4,5_)** and CA1 **(B_4,5_)**, an effect attributed largely to a CCPA-mediated change in peak input resistance (see representative traces for CA2, **A_4_**, and CA1, **B_4_**). *p < 0.05, ns, not significant.

To exclude the possibility that the synaptic depression observed in CA2 and CA1 neurons resulted from A_1_R-dependent changes in neuronal excitability induced by CCPA, we tested the effects A_1_R activation on the intrinsic membrane properties of CA2 and CA1 neurons. There was no significant effect of CCPA on the number of action potentials elicited by suprathreshold current injection or on the resting membrane potential of pyramidal neurons in CA2 (spikes, *F*_(9,120)_ = 0.03, *P* > 0.99, *n* = 7, Figures 5A_1,2_; RMP, *t*_(6)_ = 1.89, *P* = 0.108, *n* = 7, Figure 5A_3_) or CA1 (spikes, *F*_(9,100)_ = 0.36, *P* = 0.95, *n* = 6, Figures 5B_1,2_; RMP, *t*_(5)_ = 0.32, *P* = 0.759, *n* = 6, Figure 5B_3_). Interestingly, although CCPA had no significant effect on the steady-state input resistance of neurons in both CA2 and CA1 (data not shown), there was a significant reduction in the amount of inward rectification induced by CCPA in both CA2 and CA1 pyramidal cells. The rectification ratio decreased from 1.38 ± 0.07 to 1.27 ± 0.07 in area CA2 (*t*_(6)_ = 3.70, *P* < 0.05, *n* = 7, Figures 5A_4,5_) and from 1.31 ± 0.03 to 1.19 ± 0.01 in CA1 (*t*_(5)_ = 7.82, *P* < 0.001, *n* = 6, Figures 5B_4,5_) following bath-application of 100 nM CCPA. Indeed, there was a marked reduction in the “sag” present in the voltage responses to −100 pA current steps induced by CCPA in both CA2 (Figure 5A_4_) and CA1 (Figure 5B_4_) neurons. These data suggest that activation of A_1_Rs has a minimal effect on the overall excitability of principal neurons in the hippocampus and that A_1_Rs may regulate the activity of transmembrane currents responsible for inward rectification at hyperpolarized membrane potentials (perhaps *via* the hyperpolarization-activated nonspecific cation current, *I*_h_). Previously, A_1_R activation has been shown to activate G-protein-activated K^+^ channels (or GIRKS) to inhibit the activity of CA1 neurons (Trussell and Jackson, 1987; Lüscher et al., 1997; Wetherington and Lambert, 2002).

### Mechanisms Underlying the A_1_R-Mediated Synaptic Depression in CA2 and CA1

**Figure 6:**
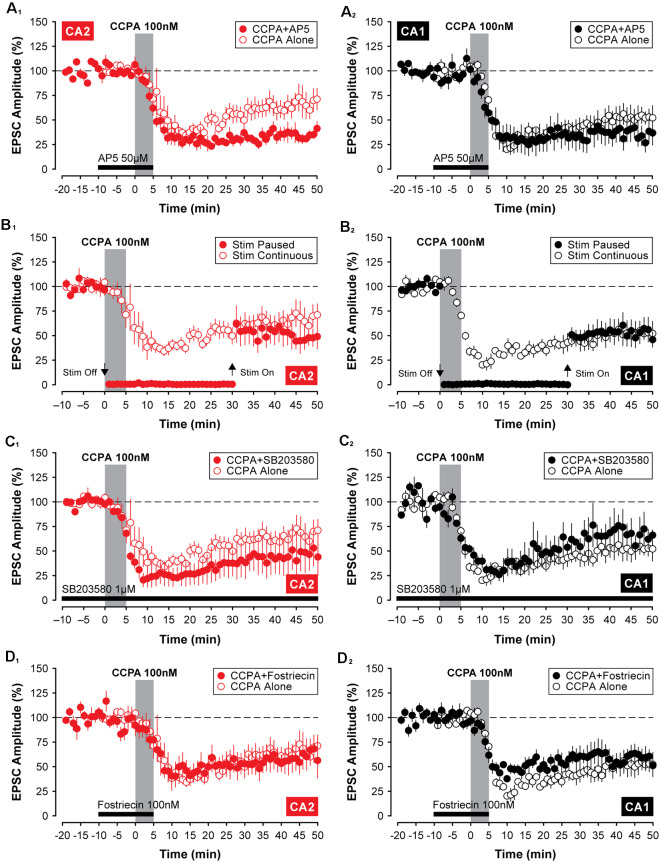
The A_1_R-mediated depression of EPSCs in CA2 and CA1 does not require activation of NMDARs, p38 MAP Kinase, or protein phosphatase 2A. Except for a significant *enhancement* of CCPA-induced depression by 50 μM AP5 in area CA2 **(A_1_)**, blockade of N-methyl-D-aspartate (NMDA) receptors, p38 MAP kinase or protein phosphatase 2A with 50 μM AP5, 1 μM SB203580 or 100 nM fostriecin (latency indicated by the black bars), respectively, did not affect the depression of EPSCs induced by 5-min bath-application of CCPA in CA2 **(A_1_,C_1_,D_1_)** or CA1 **(A_2_,C_2_,D_2_)**. Also, the constant synaptic drive was not required for the induction of A_1_R-mediated synaptic depression in the hippocampus. A temporary pause in the delivery of test stimulation to the Schaffer collaterals for 30 min during and after application of CCPA (Stim Paused) was similarly without effect in either recording site (CA2, **B_1_**; CA1, **B_2_**).

To determine whether the depression of EPSCs induced by CCPA was mechanistically similar to other forms of synaptic depression in the hippocampus, we applied treatments that have been shown to affect synaptic function in other models of synaptic plasticity. Because activity-dependent forms of long-term depression (LTD) can rely on activation of NMDA receptors (Dudek and Bear, 1992), we tested whether the NMDA receptor antagonist, AP5, would block the CCPA-mediated depression shown in Figures 2–4. Co-application of 50 μM AP5 with CCPA was not sufficient to block the A_1_R-mediated depression of EPSCs in both CA2 and CA1. Indeed, in area CA1, EPSCs were depressed significantly to 33.53 ± 10.15% of baseline when assessed during the last 5-min of the experiment (*F*_(2,14)_ = 26.78, *P* < 0.0001, *n* = 5, Figure 6A_2_). Although EPSCs were depressed by the co-application of CCPA and AP5 relative to untreated controls (Tukey *P* < 0.0001), responses did not differ significantly from slices treated with CCPA alone (Tukey *P* = 0.206). Interestingly, in area CA2, the co-application of AP5 significantly *enhanced* the depression of synaptic transmission induced by CCPA (see Figure 6A_1_). EPSCs were reduced to 34.57 ± 4.32% of baseline in slices treated with both AP5 and CCPA compared to a reduction of only 61.81 ± 9.38% for slices treated with CCPA alone (*F*_(2,15)_ = 22.66, *P* < 0.0001, *n* = 5, Tukey *P* < 0.05). It is unclear why blockade of NMDARs facilitates the CCPA-mediated suppression of EPSCs in area CA2, but activation of NMDARs is not a requirement for induction of A_1_R-mediated synaptic depression in either CA2 or CA1.

We have shown previously that constant synaptic stimulation of the Schaffer collaterals is not required for the induction of A_1_R-mediated synaptic potentiation in area CA2 (Simons et al., 2012). In the current study, the depression of EPSCs induced by CCPA was also unaffected by a temporary pause in the delivery of test stimulation during the experiment. Responses in CA2 were depressed to 47.11 ± 9.87% of baseline despite test stimulation being suspended for a 30-min period during and after bath-application of CCPA (*F*_(2,15)_ = 11.83, *P* < 0.001, *n* = 5; Figure 6B_1_). Although the amplitude of EPSCs was depressed significantly relative to untreated controls (Tukey *P* < 0.01), pausing test stimulation did not affect the magnitude of the CCPA-mediated depression-induced in CA2 once synaptic stimulation was resumed (Tukey *P* = 0.427; compared to CCPA-treated slices receiving test stimulation every 20 s). Similar results were obtained in recordings made from neurons in area CA1 (EPSCs depressed to 52.21 ± 10.30% of baseline, *F*_(2,14)_ = 17.51, *P* < 0.001, *n* = 5; control vs. Stim Paused, Tukey *P* < 0.001; Stim Paused vs. Stim Continuous, Tukey *P* = 0.999; Figure 6B_2_). These results indicate that repeated test stimulation of the Schaffer collaterals is not a prerequisite for inducing A_1_R-dependent depression of EPSCs by CCPA.

Previous work has shown that the activity of p38 MAP kinase contributes to A_1_R-mediated synaptic depotentiation (Liang et al., 2008) and depression (Brust et al., 2006) in CA1 neurons. As such, we tested whether inhibition of p38 MAP kinase would block the induction of CCPA-mediated synaptic depression in Schaffer collateral inputs to CA2 and CA1 neurons in juvenile slices. Inclusion of 1 μM SB203580 in the internal electrode solution was not sufficient to prevent the induction of A_1_R-dependent synaptic depression in the hippocampus. In area CA2, synaptic responses were significantly depressed to 48.49 ± 18.6% of baseline relative to untreated controls (*F*_(2,14)_ = 7.21, *P* < 0.01, Tukey *P* < 0.05, *n* = 4, Figure 6C_1_), and responses did not differ from those evoked in separate experiments in which CCPA was applied without the kinase inhibitor (Tukey *P* = 0.651). In contrast to previous work, inhibition of p38 MAP kinase also did not block A_1_R-mediated synaptic depression in hippocampal area CA1. EPSCs were depressed to 64.65 ± 12.09% of baseline by CCPA relative to controls (*F*_(2,12)_ = 16.78, *P* < 0.001, Tukey *P* < 0.05, *n* = 3, Figure 6C_2_), and the magnitude of the depression was similar to experiments in which CCPA was applied alone (Tukey *P* = 0.535). Thus, in slices from juvenile animals, the activity of p38 MAP kinase does not appear to play a significant role in A_1_R-dependent synaptic depression induced by 100 nM CCPA in either recording site.

There is significant data to support a key role for protein phosphatases in activity-dependent forms of LTD in the hippocampus (Mauna et al., 2011), including protein phosphatase 2A (Stockwell et al., 2015). Here, we investigated whether the inhibiting activity of protein phosphatase 2A with fostriecin would block the A_1_R-mediated synaptic depression induced by CCPA. Similar to the negative results described above for various other inhibitors, co-application of 100 nM fostriecin with CCPA did not block the A_1_R-mediated synaptic depression in either CA2 or CA1. The amplitude of EPSCs was reduced significantly to 63.19 ± 14.43% of baseline and to 58.63 ± 7.30% of baseline in CA2 and CA1, respectively, relative to untreated controls (CA2, *F*_(2,16)_ = 5.03, *P* < 0.05, *n* = 6, Tukey *P* < 0.05, Figure 6D_1_; CA1, *F*_(2,15)_ = 19.57, *P* < 0.0001, *n* = 6, Tukey *P* < 0.001, Figure 6D_2_). Further, the depression of synaptic responses in CA2 and CA1 induced by CCPA in the presence of fostriecin did not differ significantly from the depression induced by CCPA alone (CA2, Tukey *P* = 0.995; CA2, Tukey *P* = 0.789). Although we do not provide positive evidence that fostriecin was effective, we note that we used 5 times the concentration found effective for glutamate receptor internalization (Stockwell et al., 2015). Nevertheless, these data suggest that the activity of protein phosphatase 2A is not required for A_1_R-mediated synaptic depression in the hippocampus and that other A_1_R-mediated intracellular signals are likely involved.

### Divergence in A_1_R-Mediated Intracellular Signaling in CA2 and CA1 Neurons

**Figure 7:**
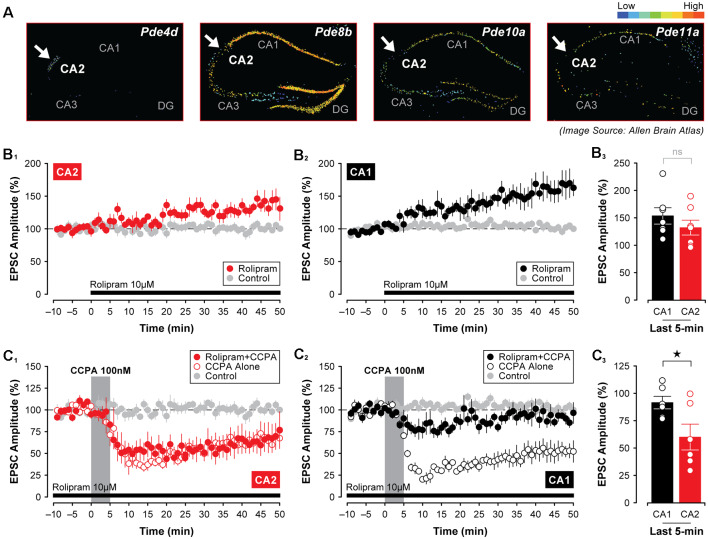
The A_1_R-mediated depression of synaptic responses in CA1 relies on phosphodiesterase activity. **(A)** The expression of several phosphodiesterases (PDEs) differs in mouse CA1 and CA2 with *Pde4d* showing the highest expression in CA2 (arrow), but with *Pde8b*, *Pde10a*, and *Pde11a* showing the lowest levels in CA2 compared to area CA1 (arrows; Lein et al., 2006). **(B_1–3_)** The PDE inhibitor rolipram (10 μM) induced a slow-onset potentiation of EPSCs that did not differ between CA1 and CA2. However, pre-treating slices with rolipram for 40 min followed by its continuous perfusion throughout the experiment blocked the CCPA-mediated depression of EPSCs in CA1, but not in CA2 **(C_1–3_)**. *p < 0.05, ns, not significant.

Adenosine A_1_Rs couple to G_i/o_ (Munshi et al., 1991; Dunwiddie and Masino, 2001), and as such, activation of A_1_Rs by selective agonists should reduce the activity of adenylyl cyclase and constrain the production of cAMP. If the CCPA-mediated synaptic depression in CA2 and CA1 is linked to an A_1_R-dependent decrease in postsynaptic levels of cAMP then the depression of EPSCs would not necessarily be expected to occur as rapidly as we observe here, even though A_1_R agonists are known to stimulate internalization of glutamate receptors over long periods (Stockwell et al., 2015). For cAMP levels to drop so precipitously, enzymatic degradation of cAMP would presumably be required. For this reason, we tested whether differences in A_1_R-mediated synaptic plasticity between areas CA2 and CA1 might reflect regional variations in phosphodiesterase activity in the hippocampus. Indeed, the expression of several isoforms of phosphodiesterase differs across hippocampal subfields in mice. Specifically, expression of *Pde8b*, *Pde10a*, and *Pde11a* is highest in area CA1, whereas *Pde4d* is expressed exclusively in area CA2 (see Figure 7A; Lein et al., 2006). Incidentally, both *Pde8b* and *Pde11a* increase significantly during aging (Kelly et al., 2014). To determine whether inhibition of phosphodiesterase activity mimics effects observed using various A_1_R antagonists on synaptic transmission in the hippocampus (i.e., induces synaptic potentiation), we bath-applied the phosphodiesterase inhibitor, rolipram, and assessed its effects on evoked synaptic responses in CA2 and CA1. Continuous application of 10 μM rolipram for 50-min induced a slight facilitation of EPSCs in CA2–132.70 ± 13.59% of baseline, but this increase did not differ significantly from control responses (*t*_11_ = 2.08, *P* = 0.061, *n* = 7, Figure 7B_1_). In CA1, however, EPSCs were facilitated to 154.10 ± 14.99% of baseline by rolipram, and this potentiation was significant relative to responses evoked in control experiments (*t*_12_ = 3.44, *P* < 0.01, *n* = 7, Figure 7B_2_). Despite this increase, the magnitude of synaptic potentiation induced by rolipram did not differ significantly between CA2 and CA1 (*t*_(12)_ = 1.06, *P* = 0.3104, Figure 7B_3_).

Interestingly, rolipram’s ability to induce a significant potentiation of EPSCs in area CA1 was coincident with its ability to block the synaptic depression induced by CCPA. For these experiments, slices were first pre-incubated in oxygenated rolipram (10 μM rolipram in normal ASCF) for at least 40 min before being transferred to the recording chamber. This pre-incubation period was then followed by the continuous perfusion of 10 μM rolipram for the entire duration of the experiment. Under these conditions, the A_1_R-mediated depression of synaptic responses typically induced by 5-min application of CCPA was blocked in Schaffer collateral inputs to CA1 (Figure 7C_2_), but not to CA2 (Figure 7C_1_). In area CA1 of rolipram-treated slices, EPSCs remained stable at 91.80 ± 5.77% of baseline when assessed during the last 5-min of recording, and responses did not differ significantly from untreated controls (*F*_(2,15)_ = 19.55, *P* < 0.0001, *n* = 6, Tukey *P* = 0.432). However, in CA2, rolipram treatment had no effect on the depression induced by CCPA (EPSCs depressed to 60.28 ± 11.79% of baseline, *F*_(2,16)_ = 6.78, *P* < 0.01, *n* = 6, Rolipram + CCPA vs. control, Tukey *P* < 0.05). In line with this, the depression of EPSCs was significantly greater in CA2 than in CA1 (*t*_(10)_ = 2.40, *P* < 0.05, Figure 7C_3_). This suggests that a mechanism other than robust phosphodiesterase activity may be at work in CA2 neurons to cause synaptic depression in response to A_1_R agonists.

**Figure 8:**
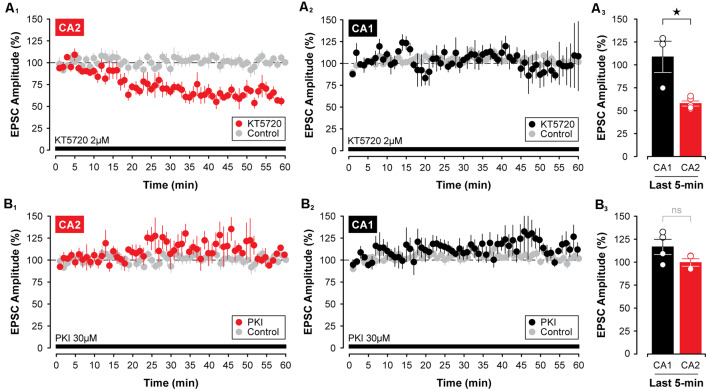
Inhibition of protein kinase A (PKA) with KT5720 induces a slow-onset depression of synaptic transmission in area CA2, but not in area CA1. **(A_1–3_)** Including the PKA inhibitor, KT5720 (2 μM, indicated by the black bar), in the intracellular recording solution caused a significant decrease in the amplitude of EPSC in CA2, but not in CA1. **(B_1–3_)** Interestingly, the internal application of the more selective inhibitor of PKA, PKI (30 μM; black bar), was without effect on EPSCs recorded in CA2 or CA1. PKI was obtained from Santa Cruz Biotechnology. *p < 0.05, ns, not significant.

Although some phosphatases, such as protein phosphatase 2A and protein phosphatase 4, are not required for the decline in synaptic strength induced by CCPA (see Figures 6D_1,2_), it is possible that blocking the activity of key protein kinases directly may be sufficient to induce a lasting depression of synaptic transmission in the hippocampus that resembles the depression mediated by activation of A_1_Rs. This depends critically, though, on whether the depression of EPSCs involves the downregulation of cAMP-dependent protein kinase activity as opposed to regulation of other cAMP-dependent proteins, such as the exchange protein activated by cAMP, or EPAC (Sugawara et al., 2016). Given that PKA is the principal kinase stimulated by the activity of cAMP, we tested two different PKA inhibitors for their efficacy in mimicking the CCPA-mediated depression of EPSCs in CA2 and CA1. Interestingly, 2 μM KT5720, but not 30 μM PKI, in the internal electrode solution, caused a slow-onset depression of synaptic responses in CA2. Indeed, synaptic responses were reduced significantly to 58.01 ± 2.77% of baseline by KT5720 in CA2 relative to untreated controls when assessed during the last 5-min of the experiment (*t*_(9)_ = 13.18, *P* < 0.0001, *n* = 5, Figure 8A_1_). Interestingly, KT5720 had no effect on the amplitude of EPSCs in CA1 (responses remained stable at 108.90 ± 17.06% of baseline, *t*_(8)_ = 0.651, *P* = 0.533, *n* = 3, Figure 8A_2_; CA2 vs. CA1, *t*_(6)_ = 3.92, *P* < 0.01, Figure 8A_3_), and inclusion of PKI in the internal electrode solution had no effect on responses irrespective of recording site (CA2, EPSCs stable at 100.50 ± 4.12%, *t*_(8)_ = 0.289, *P* = 0.780, *n* = 4, Figures 8B_1,3_; CA1, EPSCs stable at 117.60 ± 8.24%, *t*_(9)_ = 2.21, *P* = 0.054, Figures 8B_2,3_; CA2 vs. CA1, *t*_(6)_ = 1.86, *P* = 0.113, Figure 8B_3_) or concentration (10 μM, data not shown).

**Figure 9:**
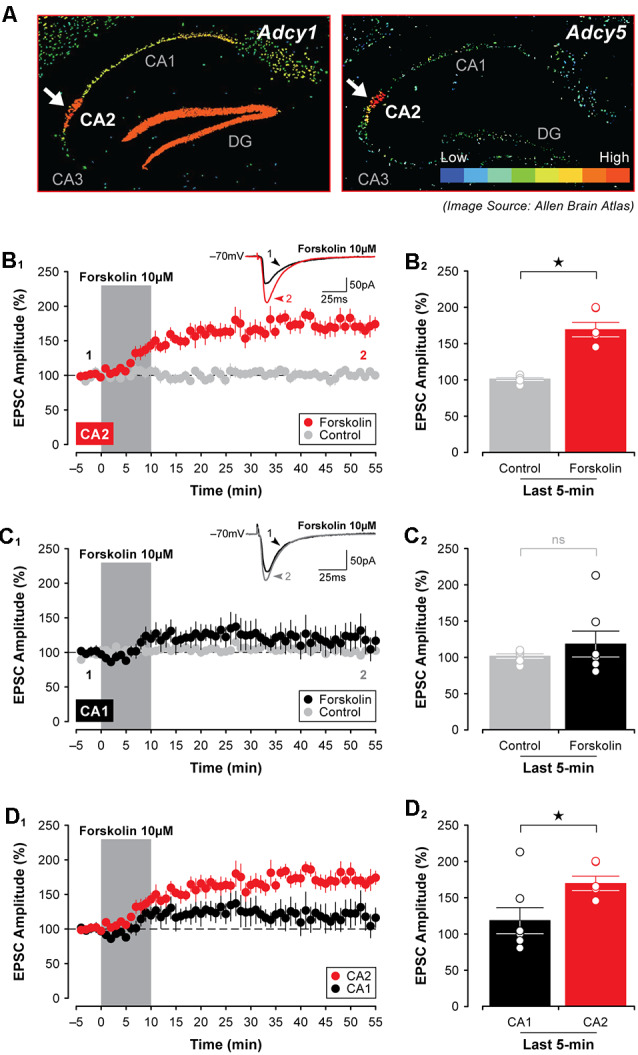
Activation of adenylyl cyclases enhances EPSCs in CA2 in juvenile brain slices. **(A)** Expression of several adenylyl cyclases is greater in CA2, including *Adcy1*, *Adcy5* (shown; arrows), and *Adcy6* (Lein et al., 2006). **(B_1,2_)** Bath-application of the adenylyl cyclase activator, forskolin (10 μM), for 10 min induced a lasting potentiation of EPSCs in area CA2 that was significantly greater than responses in age-matched and untreated control experiments. However, forskolin had no lasting effect on transmission in area CA1 **(C_1,2_)**. **(D_1,2_)** The response to forskolin was significantly greater in CA2 than in CA1 (data re-plotted from **(B_1_,C_1_**). *p < 0.05, ns, not significant.

Finally, because at least three isoforms of adenylyl cyclase are highly expressed in area CA2 in the mouse (Visel et al., 2006), including *Adcy1*, *Adcy5*, and *Adcy6* (see Figure 9A)—an effect observed even in animals as young as P7 (for *Adcy1*, Lein et al., 2006)—we tested whether direct stimulation of adenylyl cyclases could mimic the effects of A_1_R antagonists and induce synaptic potentiation in area CA2 in slices from juvenile animals. Indeed, bath application of 10 μM forskolin caused a rapid potentiation of EPSCs in CA2 neurons that persisted for the entire duration of the experiment. EPSCs were potentiated to 170.20 ± 10.16% of baseline (Figure 9B_1_, *n* = 6), and this facilitation was significant relative to responses in age-matched and untreated controls (*t*_(10)_ = 6.62, *P* < 0.0001, Figure 9B_2_). As predicted based on our work previously (Simons et al., 2012), there was no effect of forskolin on responses in CA1 neurons (responses remained stable at 118.60 ± 17.89% of baseline, *t*_(11)_ = 0.866, *P* = 0.405, *n* = 7, Figures 9C_1,2_). Not surprisingly, the magnitude of the synaptic potentiation induced by forskolin was significantly greater in CA2 than in CA1 (*t*_(11)_ = 2.39, *P* < 0.05, Figures 9D_1,2_). Taken together, these data suggest that although A_1_R expression does not differ substantially across the hippocampus at early postnatal ages, the activity of critical A_1_R-dependent intracellular signals—including adenylyl cyclase, protein kinase, and phosphodiesterase activity—differ considerably between CA1 and CA2, thus resulting in an overall effect that essentially mimics the adult state.

## Discussion

In this study, we pursued two lines of inquiry related to adenosine receptor expression in the hippocampus. First, we tested whether the use of a selective A_1_R agonist to induce synaptic depression in CA2 and CA1 would better reflect changes in the staining pattern of A_1_Rs in the hippocampus than the application of A_1_R antagonists, such as caffeine or DPCPX. Second, we tested several of the known intracellular signals linked to activation of A_1_Rs and whether they might account for the dramatic effects of A_1_R antagonists observed in CA2 neurons in slices prepared from young animals (Simons et al., 2012). Indeed, both lines of inquiry produced data to support the findings of Ochiishi et al. (1999) in that—at least in Sprague–Dawley rats—A_1_Rs in the hippocampus are distributed evenly across areas CA2 and CA1 in juvenile animals, and that A_1_R expression in CA2 neurons increases markedly during typical adolescent development (see Figure 3). Our study also highlights critical intracellular signals downstream to activation of A_1_Rs that contribute to the robust effects of adenosine receptor antagonists observed in area CA2 during early postnatal development, which occur before any enrichment of A_1_Rs in CA2 pyramidal neurons.

Adenosine receptors localized in presynaptic boutons are known to regulate neurotransmitter release (Prince and Stevens, 1992; Wetherington and Lambert, 2002; Scammell et al., 2003), but they also play critical roles in mediating the activity of key postsynaptic signaling cascades and in controlling intrinsic membrane conductances that affect neuronal excitability (Greene and Haas, 1985; Gerber et al., 1989). Here, we observed several such effects following bath application of the selective A_1_R agonist CCPA that do not differ between areas CA2 and CA1, including changes in rectification ratios and synaptic function (see Figures 2, 5, respectively) that are similar to those reported previously (Greene and Haas, 1991; Dunwiddie and Masino, 2001). It is unclear, however, whether the lasting effects of CCAP on synaptic and intrinsic excitability shown in the current study are due to A_1_Rs located pre- or postsynaptically in CA2 and CA1 (but see Figure 4). Notably, though, adenosine receptors have been shown to interfere with postsynaptic mechanisms required for the maintenance of activity-dependent forms of LTP in area CA1 (Arai et al., 1990; Rex et al., 2005). The possibility of reversing LTP in CA2 neurons by subsequent activation of A_1_Rs, however, was not tested in the current study because LTP of synaptic responses rarely occurs in Schaffer collateral inputs to CA2 when performing typical whole-cell slice experiments *in vitro* with 2 mM extracellular calcium in the bathing medium (Zhao et al., 2007; Simons et al., 2009; Chevaleyre and Siegelbaum, 2010). Our data presented here do not rule out a role for A_1_Rs regulating GABAergic transmission, such as has been described previously (Jeong et al., 2003; Rombo et al., 2014), but we note that Muñoz and Solís (2019) found no effect of picrotoxin on CA1/CA2 differences in caffeine-induced potentiation. This is particularly relevant in light of the findings that CA2 of rats and mice has a higher density of some types of GABAergic interneurons than CA1 (Piskorowski and Chevaleyre, 2013; Botcher et al., 2014).

Regarding the effects of A_1_R activation on presynaptic terminals, our primary evidence against the idea of a CA1/CA2 difference in presynaptic neurotransmitter release is based on our analysis of PPF. This technique presumes that the identical terminals activated by the first pulse are also activated by the second, and so neurotransmitter release may be underestimated in our experiments to a large degree. We note that although we did not see significant effects of CCPA (A_1_R agonist) on PPF or differences between CA1 and CA2 in slices from juvenile animals, Muñoz and Solís (2019) recently reported that the magnitude of PPF differed between CA1 and CA2 after caffeine (A_1_R antagonist) treatment in slices from older animals. The high variance in synaptic responses seen with whole-cell recordings compared with the low variance in responses recorded in field potentials (Muñoz and Solís, 2019) may have also contributed to the differences in findings in the two studies. Additional experiments with more cells may reveal significant findings, but the lack of differences between CA1 and CA2 in: (a) A_1_R staining at P14 (Ochiishi et al., 1999), and (b) CCPA-induced depression (Figure 3) at P14 is consistent with our findings of modest/variable changes in PPF. Selective disruption of G_i/o_ coupling in the recorded neuron or localized knockout of *Adora1* may eventually resolve the question of the presynaptic effects of adenosine.

Although the developmentally-regulated pattern of immunolabeling for the A_1_R in area CA2 first shown by Ochiishi et al. (1999) has not been replicated successfully by us or by others using commercially-available antibodies (Rex et al., 2005), our finding that an A_1_R agonist was sufficient to cause an age-dependent change in synaptic function in both CA2 and CA1 directly supports the idea of a developmental shift in the distribution of A_1_Rs across the hippocampus (see Figure 3). Interestingly, previous immunolabeling work has shown that 5’-nucleotidase—an enzyme required to generate adenosine from ATP—is highly enriched in area CA2 in both the mouse and the gerbil (Lee et al., 1986). Nevertheless, it had yet to be reconciled how a blockade of A_1_Rs by caffeine or another A_1_R antagonist could induce a robust potentiation of synaptic responses in area CA2 at concentrations that do little in CA1 and CA3 (Simons et al., 2012). Here, we provide evidence showing how several A_1_R-mediated intracellular signals differ between areas CA2 and CA1, and this suggests that differences in A_1_R-mediated synaptic plasticity observed in slices prepared from juvenile animals may be due to mechanisms other than those linked directly to the expression pattern of A_1_Rs in the hippocampus. Specifically, both kinase activity, required for maintaining synaptic responses (see Figure 8), and robust response to forskolin in CA2 neurons (see Figure 9) indicate that postsynaptic currents in CA2 pyramidal cells are especially sensitive to manipulations of cAMP and changes in substrate phosphorylation, even in tissue obtained from young animals. Alternatively, rolipram-sensitive phosphodiesterase activity, which is required for A_1_R-mediated synaptic depression in CA1 but not in CA2 (see Figure 7), may be responsible for the blunted potentiating effects of forskolin and various A_1_R antagonists in area CA1. Importantly, though, the two are not likely to be mutually exclusive and both may contribute to the relative effectiveness of A_1_R antagonists to potentiate EPSCs in slices from young rats. Regional differences in the expression of A_1_Rs may be sufficient to explain the differential effects of CCPA on synaptic responses in adult tissue, but they would likely be in addition to any regional differences in intracellular signaling molecules described above. For example, both of the calcium-activated adenylyl cyclases, AC1 and AC8, increase with age in mouse hippocampus (Nicol et al., 2005; Conti et al., 2007). In addition, the antagonist and agonist responses may be controlled by adenosine re-uptake and metabolism pathways, any of which may also be developmentally controlled. Thus the CPPA response, presuming it is neither taken up or metabolized, may reflect partial occlusion by these other factors regulating adenosine tone; caution must be taken when interpreting these data.

The precise mechanism underlying the induction of CCPA-mediated synaptic depression in Schaffer collateral inputs to CA2 and CA1 remains unknown, but it is clear that NMDA receptors or constant synaptic stimulation are not required (see Figures 6A_1,2_,B_1,2_). These findings indicate that mechanisms typically involved in the induction of activity-dependent forms of synaptic depression in the hippocampus are not required here [but see Pagani et al. (2014) for mechanisms of Avpr1b-mediated synaptic potentiation in CA2], and this is consistent with the findings of Simons et al. (2012) who demonstrated a role for enhanced activity of PKA in the expression of A_1_R-mediated synaptic potentiation in area CA2 induced by caffeine or DPCPX (Simons et al., 2012). Also, previous work examining the effects of the A_1_R agonist CPA on glutamate receptor phosphorylation and internalization indicates a key role for protein phosphatase 2A (Stockwell et al., 2015), which has also been implicated in activity-dependent forms of LTD (Mauna et al., 2011). Although we used a different A_1_R agonist for a much shorter incubation period than in the previous report, we found no effect of the protein phosphatase 2A inhibitor, fostriecin, on the CCPA-mediated depression of EPSCs in either CA2 or CA1 (see Figures 6D_1,2_). We also found that inhibition of p38 MAP kinase, which has been implicated in both adenosine-mediated signaling and NMDA receptor-dependent LTD (Bolshakov et al., 2000; Brust et al., 2006; Liang et al., 2008), was similarly without effect (see Figures 6C_1,2_). Thus, although there are several forms of LTD (Sanderson et al., 2016; Pinar et al., 2017), many of which have underlying mechanisms shared with adenosine receptor-mediated reductions in synaptic currents, our data suggest that NMDARs, phosphatase 2A, or p38 MAP kinase are unlikely to be critical for the type CCPA-induced depression reported here. Further studies would be required to determine whether other mechanisms associated with pre- and/or post-synaptic forms of LTD, such as those requiring endocannabinoids or mGluRs, interact with A_1_R-mediated depression (Atwood et al., 2014; Kano, 2014; Olmo et al., 2016).

A difficult result to interpret is the observation that internal application of the different protein kinase A inhibitors elicited different effects across the various hippocampal subfields (KT5720 and PKI; see Figures 8A,B, respectively). Specifically, KT5720 caused a slow-onset decay in the amplitude of EPSCs recorded in CA2 neurons, yet it had no effect on transmission in CA1 (see Figures 8A_1–3_). Interestingly, PKI in the internal electrode solution did not affect synaptic responses evoked in either recording site (see Figures 8B_1–3_). Based on the findings of Kameyama et al. (1998) who observed a rundown of responses in CA1 with 10 μM PKI, we had expected a similar result here. However, our initial attempts with 10 μM were without effect (data not shown), and even a 30 μM concentration of PKI failed to induce changes in synaptic transmission after 1 h of recording (see Figures 8B_1,2_). Although possibly the rundown observed with PKI in the previous study was due to the intracellular recording technique (i.e., sharp intracellular recordings using an internal solution containing only salts), we did obtain the expected result with a different PKA inhibitor using a different slice recording protocol (KT5720; whole-cell patch-clamp recordings with ATP and GTP in the internal solution, see Figure 8A_1_). Indeed, the two inhibitors are known to act in slightly different ways to inhibit the activity of PKA, with PKI binding the catalytic subunits of the kinase and KT5720 acting as a competitive antagonist at the ATP binding sites on the catalytic domains (Murray, 2008). KT5720 is thought to be the less selective inhibitor out of the two, raising the possibility that its effects on synaptic responses in CA2 neurons may be due actions on other intracellular kinases, such as MEK or MAPK (Murray, 2008). Alternatively, if the PKA is bound together in complexes, such as AKAPs (Nygren and Scott, 2015), then the PKI binding site may not be accessible to exogenously applied compounds. Nevertheless, we believe that the results here indicate that various targets of PKA, including glutamate receptors, may be differentially regulated in both CA2 and CA1, and this may partly explain the robust potentiating effects of A_1_R antagonists in area CA2 in slices obtained from young animals despite the lack of any obvious enrichment of A_1_Rs.

Hippocampal area CA2 has only recently been appreciated for its role in various forms of cognition. Indeed, evidence has been amassed linking CA2 to a wide array of social behaviors. Specifically, lesioning area CA2 or silencing CA2 neurons *via* genetic manipulations to prevent glutamate release results in deficits in social recognition memory (Hitti and Siegelbaum, 2014; Stevenson and Caldwell, 2014), deletion of a CA2-enriched gene, *Avpr1b*, reduces aggressive behavior in mice (Wersinger et al., 2002; Pagani et al., 2014) and optogenetic stimulation of vasopressinergic axons in CA2 enhances social recognition memory (Smith et al., 2016). Although A_1_Rs are expressed in neurons throughout the brain, if their function is greatest in area CA2 where the receptor is highly concentrated then it is not surprising that an A_1_R agonist *impairs* social recognition memory and that caffeine *enhances* it (Prediger and Takahashi, 2005). Moreover, A_1_R knockout mice display no deficits in spatial learning, but they do exhibit pronounced anxiety-like behaviors and aggression (Giménez-Llort et al., 2002, 2005). Finally, chronic administration of caffeine increases aggressive behavior in rats (Fredholm et al., 1999) and enhances both the length and branching pattern of basal dendrites in CA1, including the density of spines on these dendrites (Vila-Luna et al., 2011) which are the main targets of CA2 neurons (Kohara et al., 2013). Thus, links between adenosine receptor function, various social behaviors, and area CA2 may be worthy of further consideration. Our findings indicate that downstream signaling of A_1_Rs is what distinguishes CA2 from CA2 early in postnatal development. Further, they suggest that the modulation of key neurotransmitter systems in the hippocampus, like adenosine, may differ depending on the stage of development.

## Data Availability Statement

The raw data supporting the conclusions of this article will be made available by the authors, without undue reservation, to any qualified researcher.

## Ethics Statement

The animal study was reviewed and approved by the National Institute of Environmental Health Sciences Animal Care and Use Committee.

## Author Contributions

SD and DC conceived and designed the studies and wrote the manuscript. DC conducted experiments and analyzed data. SD supervised the project.

## Conflict of Interest

The authors declare that the research was conducted in the absence of any commercial or financial relationships that could be construed as a potential conflict of interest.

## Abbreviations

Adenosine: 9-β-D-ribofuranosyl-9*H*-purin-6-amine
AP5: 2-amino-5-phosphonovaleric acid
CCPA: 2-chloro-*N*-cyclopentyladenosine
Forskolin: [3*R*-(3α,4aβ,5β,6β,6aα,10α,10aβ,10bα)]-5-(Acetyloxy)-3-ethenyldodecahydro-6,10,10b-trihydroxy-3,4a,7,7,10a-pentamethyl-1*H*-naphtho[2,1-*b*]pyran-1-one
Fostriecin: (6*R*)-5,6-dihydro-6-[(1*E*,3*R*,4*R*,6*R*,7*Z*,9*Z*,11*E*)-3,6,13-trihydroxy-3-methyl-4-(phosphonooxy)-1,7,9,11-tridecatetraenyl]-2*H*-pyran-2-one
KT5720: (9*R*,10*S*,12*S*)-2,3,9,10,11,12-hexahydro-10-hydroxy-9-methyl-1-oxo-9,12-epoxy-1*H*-diindolo[1,2,3-*fg*:3′,2′,1′-*kl*]pyrrolo[3,4-*i*][1,6]benzodiazocine-10-carboxylic acid
PKI: Protein Kinase A inhibitor fragment (6–22) amide Thr-Tyr-Ala-Asp-Phe-Ile-Ala-Ser-Gly-Arg-Thr-Gly-Arg-Arg-Asn-Ala-Ile-NH_2_
Rolipram: 4-[3-(cyclopentyloxy)-4-methoxyphenyl]pyrrolidin-2-one
SB203580: 4-[5-(4-fluorophenyl)-2-[4-(methylsulfonyl)phenyl]-1*H*-imidazol-4-yl]pyridine.
